# Wheal and flare reactions in skin prick tests of patients treated with montelukast alone or in combination with antihistamines

**DOI:** 10.1007/s00011-013-0688-y

**Published:** 2013-11-27

**Authors:** Malgorzata Gorska Ciebiada, Marcin Barylski, Maciej Ciebiada

**Affiliations:** 1 Department of Internal Medicine and Diabetology, Medical University of Lodz, Lodz, Poland; 2 Department of Internal Disease and Cardiological Rehabilitation, Medical University of Lodz, Lodz, Poland; 3 Department of General and Oncological Pneumonology, Medical University of Lodz, Kopcinskego Street 22, 90-153 Lodz, Poland

**Keywords:** Allergic rhinitis, Montelukast, Antihistamine, Skin prick test, Inflammation

## Abstract

**Background:**

Because antileukotrienes may inhibit inflammation, it is plausible that montelukast administered for a long time could suppress skin wheal and flare reaction, and thus, it should be discarded prior to the tests. This study assessed the effect of long-lasting treatment with montelukast alone or in combination with antihistamines on wheal and flare in skin pricks tests (SPT) in patients sensitized to perennial allergens.

**Methods:**

We conducted a 32-week, double-blind, placebo-controlled, cross-over and randomized trial that implicated two arms: arm A, 20 patients received levocetirizine, montelukast with or without levocetirizine or placebo; arm B, 20 patients received desloratadine, montelukast with or without desloratadine or placebo. All treatment periods lasted 6 weeks and were separated by 2-week washouts. At baseline and on the last day of each treatment period, SPT were performed in all participants.

**Results:**

Both levocetirizine and desloratadine in monotherapy, or in combination with montelukast, were effective in reducing wheal and flare in SPT. Monotherapy with montelukast did not change the size of the wheal for either histamine or for house dust mites, in either arm of the study, but significantly reduced the size of flare for histamine in arm A. Addition of montelukast to antihistamine did not exceed efficacy of monotherapy with antihistamine in both arms of the study.

**Conclusions:**

Since the size of wheal determines the results of SPT, montelukast, even taken for a long time, does not have to be discarded prior to the tests.

## Introduction

Skin prick tests (SPT) are commonly used to confirm sensitization to a wide spectrum of environmental allergens. SPT help to diagnose the underlying cause of rhinitis, asthma or urticaria, and are required to recommend appropriate prophylaxis or for qualification for immunotherapy [1, 2]. Since early wheal and flare reactions result mainly from histamine released from degranulating mast cells, it is obvious that antihistamines are more or less able to inhibit this reaction [3].

Since montelukast, a potent and selective leukotriene receptor antagonist, suppresses allergic inflammation [4–8], improves control of asthma [8] and reduces symptoms of seasonal [4, 5] and perennial [6, 7] allergic rhinitis (AR), it is possible that it may inhibit skin response to allergens measured in skin prick tests. Although guidelines for skin prick testing do not recommend discontinuation of montelukast before the SPT [1, 2], most studies relied on assessment of wheal and flare reactions after the single dose [9] or a very short-term treatment with montelukast [10]. Furthermore, there were studies that confirmed gradually increasing improvement of AR symptoms in the course of long-lasting treatment with montelukast [7, 11, 12] or combination of montelukast with antihistamine [11]. Therefore, it is plausible that one dose or short treatment with montelukast may not affect SPT, whereas long lasting treatment with this agent administered alone or in combination with antihistamine due to the increasing efficacy and immunomodulative properties may affect the skin response in SPT.

In this study, we aimed to determine the influence of montelukast administered for 6 weeks as monotherapy or added to antihistamine on the size of wheal and flare in SPT of patients with allergic rhinitis sensitized to perennial allergens, in relation to placebo as well as to monotherapy with desloratadine or levocetirizine.

### Study design

This study was designed as a prospective, double-blind, randomized, cross-over and controlled with placebo, including two arms with a 2-week run-in period and four treatment periods each lasting for 6 weeks separated by 2-week washouts.

Patients were recruited in our outpatient clinic over 4 months (June–September) and study was performed between September and March.

Study included male and female patients, aged 18–65 years with at least 2 years history of mild to severe persistent allergic rhinitis who were sensitized to perennial allergens relevant to Central Europe (HDM: house dust mite, cat and dog), confirmed by a positive history and positive results of skin prick tests, whereas patients who suffered from skin diseases that prevented execution and interpretation of skin prick tests, who were treated with systemic steroids or immunomodulative medicaments, as well as patients who were current smokers, with infection within 6 weeks preceding the study or with neoplasmatic disease and severe diseases, were excluded from the study. Pregnant and breast-feeding women were excluded too. Patients could not use an allergen-specific immunotherapy or any anti-allergic medications during the course of the study except the study medication. Xylomethasoline (0.1 %) nasal drops were allowed as a rescue medication.

After a two-week run-in period, all eligible patients (30 female, 10 male, mean age was 28.9 ± 2.7) were assigned randomly into group A (*n* = 20) receiving either levocetirizine (5 mg tablet one daily in the evening) or montelukast (10 mg tablet once daily in the evening) or a combination of montelukast and levocetirizine (in the evening) or placebo, or to group B (*n* = 20) receiving either desloratadine (10 mg tablet once daily in the evening) or montelukast (10 mg tablet once daily in the evening), or a combination of montelukast and desloratadine (in the evening) or placebo (5 mg saccharose in starch pills, one daily in the evening). Medications were administered in a cross-over and blinded manner.

Both at baseline and on the last day of each treatment period, skin prick tests were done for each participant.

All patients signed written informed consent and the study protocol was approved by the ethical committee of the Medical University in Lodz.

The principal endpoint of this study was the size of wheal and flare in skin prick tests after the 6 weeks of treatment, either with monotherapy with antihistamine (desloratadine or levocetirizine) and montelukast, or with combination therapy, which included antihistamine and montelukast in relation to the baseline test and placebo.

### Skin prick tests

Skin prick tests with 11 common allergens (Allergopharma J. Ganzer KG, Reinbeck, Germany) were performed for each patient, with histamine (10 mg/ml) as a positive and diluent as a negative control. Results were regarded as positive when the mean wheal diameter (assessed as a sum of the largest diameter and its perpendicular measurement) was greater than or equal to 3 mm. Since all patients were sensitized to the house dust mites, results of the SPT were presented for *Dermatophagoides pteronyssinus* and *farinae* in relation to the histamine.

### Statistical methods

The distribution of the results was determined with the Shapiro–Wilk normality test, while a Mann–Whitney test was used to compare groups and one-way analysis of variance (Anova) was done to compare results in each arm on different visits. A *p* < 0.05 was considered as statistically significant. The mean with standard error of the mean (SEM) was provided. Statistica 5.1 PL for Windows software (StatSoft Polska, Cracow, Poland) was used for analyses.

## Results

All randomized patients completed four treatment periods, and only two patients were lost to follow-up. All participants were sensitized to house dust mites and six patients were additionally sensitized to cat and dog allergens. Although sensitization to seasonal allergens was present in some patients, it was not essential in relation to HDM. Patients’ baseline characteristics are presented in Table 1.

**Table 1:** Patients’ baseline characteristics

	A Montelukast/levocetirizine arm	B Montelukast/desloratadine arm
Number of subjects	20	20
Mean age	23.65 ± 2.1	34.1 ± 2.69
Sex F:M	14:6	16:4
Ethnic origin	Caucasian (100 %)	Caucasian (100 %)
Duration of persistent allergic rhinitis (years)	5.65 ± 0.85	7.85 ± 1.32
Severity of AR (according to ARIA)	Moderate/severe	Moderate/severe

Data are expressed as mean value ± SEM

**Fig. 1:**
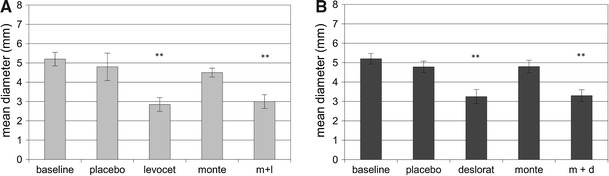
Mean diameter of wheal for histamine in patients treated in montelukast/levocetirizine arm (graph A), and montelukast/desloratadine arm (graph B). Data are expressed as mean ± SEM; m + l, montelukast with levocetirizine; m + d, montelukast with desloratadine; **p < 0.01

**Fig. 2:**
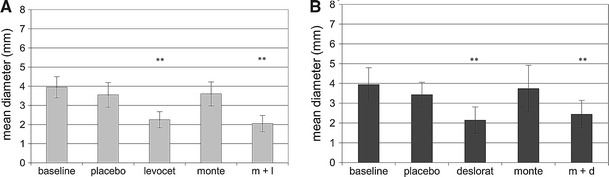
Mean diameter of wheal for *Dermatophagoides pteronyssinus* in patients treated in montelukast/levocetirizine arm (graph A), and montelukast/desloratadine arm (graph B). Data are expressed as mean ± SEM; m + l, montelukast with levocetirizine; m + d, montelukast with desloratadine; **p < 0.01

**Fig. 3:**
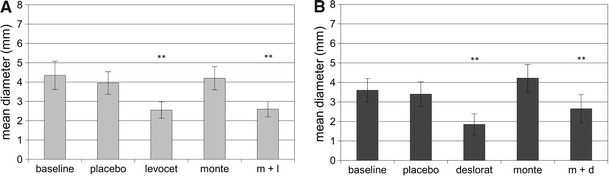
Mean diameter of wheal for *Dermatophagoides farinae* SPT in patients treated in montelukast/levocetirizine arm (graph A), and montelukast/desloratadine arm (graph B). Data are expressed as mean ± SEM; m + l, montelukast with levocetirizine; m + d, montelukast with desloratadine; **p < 0.01

Generally, the mean size of the wheal and flare was the biggest at baseline. The placebo did not affect the size of skin reactions both to histamine and HDM in patients evaluated in the arm A and B (Table 2; Figs. 1, 2, 3).

**Table 2:** Flare reaction in patients treated with: montelukast alone; levocetirizine alone; placebo or with the combination of montelukast and levocetirizine (group A) and montelukast alone; desloratadine alone; placebo or with the combination of montelukast and desloratadine (group B)

	Histamine	*D. pteronyssinus*	*D*. *farinae*
A			
Baseline	13.35 ± 1.88	11.2 ± 2.71	13.9 ± 2.9
Placebo	11.3 ± 1.66	11.25 ± 2.79	12.9 ± 2.5
Levocetirizine	2.05 ± 0.67**	4.6 ± 1.35**	4.75 ± 1.67**
Montelukast	8.95 ± 1.65*	11.5 ± 2.74	14.55 ± 2.73
Montelukast + levocetirizine	2.75 ± 0.58**	5.15 ± 1.89**	5.5 ± 1.84**
B			
Baseline	13.1 ± 1.53	10.6 ± 2.9	10.75 ± 2.53
Placebo	11.65 ± 1.73	8.55 ± 2.42	9.25 ± 2.28
Desloratadine	3.7 ± 1.2**	5.6 ± 2.2**	4.45 ± 1.8**
Montelukast	13.26 ± 1.8	9.35 ± 2.46	11.1 ± 2.46
Montelukast + desloratadine	4.4 ± 1.32**	6.1 ± 2.13**	5.75 ± 2.02**

Values ± SEM

*n* Number of subjects

* *p* < 0.05 vs. baseline

** *p* < 0.01 vs. baseline, placebo and montelukast

Levocetirizine administered as monotherapy or in combination with montelukast in arm A and monotherapy with desloratadine, as well as concomitant treatment with desloratadine and montelukast in arm B were the most effective treatment options of inhibiting the size of wheal and flare in SPT. There were no significant differences between antihistamine taken alone or in combination with montelukast (Table 2; Figs. 1, 2, 3).

If montelukast was administered as monotherapy, it did not change the size of the wheal for either histamine or for HDM, in either arm of the study. However, montelukast significantly reduced the size of flare in the SPT with histamine in arm A and slightly, but not significantly (*p* = 0.052), reduced the size of flare for *D. pteronyssinus* in arm B. Administration of montelukast in combination with antihistamine had no effect on the size of wheal and flare in comparison to monotherapy with antihistamine in both arms of the study (Table 2; Figs. 1, 2, 3).

## Discussion

The results of this study demonstrate that long-term therapy with montelukast, which is administered in monotherapy or concomitantly with the levocetirizine or desloratadine, does not affect formation of wheals in SPT, nor does it potentiate the inhibitory effect of antihistamines. Since the diameter of the wheal underlies the assessment of results of SPT, slight inhibition of flare by montelukast does not affect outcomes of the test; thus montelukast, even administered for a long time, does not have to be discarded before the skin prick test.

The formation of wheals and flares in skin prick tests result from an immunoglobulin E-dependent basophils and mast cells activation, marked by the release of inflammatory mediators, including histamine [1]. Histamine induces capillary dilation, increases vascular permeability, stimulates nociceptors responsible for pain, and further causes eosinophil chemotaxis to the inflamed tissue. As a result, the exudation enters the skin and causes swelling accompanied by itching [1, 2]. Although it is unlikely that montelukast, a potent leukotriene receptor antagonist, directly affects release of histamine from basophils and mast cells [10, 13], it is well documented that it possesses other anti-inflammatory properties [14–18], and may increase the clinical effect during the treatment [11, 12]. Thus, if taken for a long time, it could alter the inflammatory response of the skin.

Montelukast has been able to modify the skin response; however, the number of studies supporting this finding is still obscure. In rats exposed to water avoidance stress, 5-day treatment with montelukast decreased the number of both degranulated and mature granulated mast cells in the dermis [19]. In humans, montelukast significantly delayed the occurrence of late skin responses, which constitute very frequent side effects of specific immunotherapy [9], and decreased the severity of severe hypersensitivity reactions to platinum in patients undergoing rapid desensitization [20]. In patient with delayed pressure urticaria, addition of montelukast to antihistamine resulted in better improvement regarding the suppression of the challenge test and clinical improvement [21].

Despite its anti-inflammatory properties and clinical efficacy, a single dose [9] or 1-week treatment with montelukast [10, 22] did not significantly suppress both wheal [9, 10, 22] and flare [9, 10] at any time point compared with placebo. Furthermore, in this field, the efficacy of short treatment with montelukast was always inferior to efficacy of antihistamines [9, 10]. What is more, montelukast as add-on therapy to antihistamine did not bring any additional benefits compared to monotherapy with antihistamine [10].

Similarly in this study, the long-lasting treatment with montelukast did not alter the size of the wheal in SPT. The efficacy of montelukast was comparable to placebo, and significantly lower than efficacy of monotherapy with levocetirizine and desloratadine in arm A and B, respectively. Furthermore, montelukast did not change the mean diameter of flare for HDM in arm A and B and histamine in arm B, but significantly reduced the size of the flare to histamine in arm A when compared to baseline. However, the efficacy of montelukast was lower than the efficacy of levocetirizine and desloratadine and comparable to placebo at any time point of the study. Also, addition of montelukast did not potentiate effects of antihistamines.

The results of our study confirm that montelukast, which may modify tissue infiltration and inflammatory milieu, and subsequently may modify the late phase of allergic inflammation, does not seem to affect release of histamine from activated mast cells and basophils. Therefore, montelukast, even if is taken for a long time, does not need to be discontinued before allergen skin testing. The reduction of flare for histamine in patients treated with montelukast results from a small number of participants, rather than anti-inflammatory properties of montelukast, and does not affect reading of SPT where the mean size of the wheal is important.
